# Advances in attenuating opioid-induced respiratory depression: A narrative review

**DOI:** 10.1097/MD.0000000000038837

**Published:** 2024-07-19

**Authors:** Yong-Zheng Fan, Yun-Li Duan, Chuan-Tao Chen, Yu Wang, An-Ping Zhu

**Affiliations:** a The 991st Hospital of Joint Logistic Support Force of People’s Liberation Army, Xiangyang, China; b Xiangyang No. 4 Middle School Compulsory Education Department, Xiangyang, China; c Taihe Country People’s Hospital·The Taihe Hospital of Wannan Medical College, Fuyang, China.

**Keywords:** analgesia, bias agonists, naloxone, opioids, respiratory depression

## Abstract

Opioids exert analgesic effects by agonizing opioid receptors and activating signaling pathways coupled to receptors such as G-protein and/or β-arrestin. Concomitant respiratory depression (RD) is a common clinical problem, and improvement of RD is usually achieved with specific antagonists such as naloxone; however, naloxone antagonizes opioid analgesia and may produce more unknown adverse effects. In recent years, researchers have used various methods to isolate opioid receptor-mediated analgesia and RD, with the aim of preserving opioid analgesia while attenuating RD. At present, the focus is mainly on the development of new opioids with weak respiratory inhibition or the use of non-opioid drugs to stimulate breathing. This review reports recent advances in novel opioid agents, such as mixed opioid receptor agonists, peripheral selective opioid receptor agonists, opioid receptor splice variant agonists, biased opioid receptor agonists, and allosteric modulators of opioid receptors, as well as in non-opioid agents, such as AMPA receptor modulators, 5-hydroxytryptamine receptor agonists, phosphodiesterase-4 inhibitors, and nicotinic acetylcholine receptor agonists.

## 1. Introduction

Opioids are potent analgesics derived from papaver somniferum that are widely used for moderate-to-severe postoperative pain and induction of anesthesia. However, they often have side effects, such as respiratory depression (RD), tolerance, addiction, and constipation, with a perioperative RD rate of 2%. Although current clinical monitoring can reduce the risk of RD in postoperative patients, there are still clinical reports of opioid overdoses or abuse leading to death.^[1,2]^ Over the past 15 years, deaths from RD caused by widespread abuse of opioids have increased exponentially, creating a serious public health crisis,^[3]^ particularly with a surge in opioid overdoses during the COVID-19 pandemic^.[4]^ However, RD does not occur only with abuse, but also in hospitalized patients,^[5]^ individuals taking opioids for chronic pain,^[6]^ and high-risk patients, such as those with morbid obesity, sleep apnea, chronic heart failure, or certain neuromuscular diseases or at extreme ages (elderly or preterm infants).^[5]^ Therefore, improving RD is important to address opioid safety crises. Opioid-specific antagonists such as naloxone remain the preferred treatment for opioid-induced respiratory depression (OIRD) in perioperative and emergency settings; however, in some cases, naloxone may have limitations: naloxone causes loss of analgesia, withdrawal, and agitation, inducing a surge in sympathetic nerve activity, which may lead to pulmonary edema, arrhythmia, hypertension, and cardiac arrest in opioid-tolerant individuals and postoperative patients experiencing severe pain and stress; naloxone does not reverse the combination or abuse of opioids with other central nervous system inhibitors (e.g., alcohol, benzodiazepines, antidepressants, or antipsychotic drugs), leading to enhanced RD; and naloxone eliminates the half-life of approximately 30 minutes, metabolizes rapidly, and may not reverse the recurrence of RD with excessive use of potent, high-affinity, and long-acting opioids (carfentanil or high-dose fentanyl).^[7]^ For these reasons, there has been increasing interest in recent years in the development of new reversal strategies aimed at providing near-naloxone reversal of RD while retaining opioid analgesic properties, mainly focusing on 2 strategies: the development of novel opioids with weak RD, and respiratory stimulant drugs that can be administered in combination with opioids.

## 2. Intracellular mechanisms of opioid receptors

Opioid receptors belong to the G protein-coupled receptor family, which mainly includes the mu opioid receptor (MOR), kappa opioid receptor (KOR), delta opioid receptor (DOR), and nociceptin/orphanin FQ (N/OFQ) peptide receptor.^[8]^ They are widely distributed in the central nervous system, brainstem, spinal cord dorsal horns, and peripheral sites, such as the digestive tract, heart, and immune system, and are typically activated by endogenous opioid peptides (e.g., endorphins and enkephalin) to control pain and stress and regulate reward, mood, breathing, and constipation, among other physiological activities.^[9]^ After opioid receptor activation, the G protein α-subunit dissociates from the βγ subunit complex (G_βγ_), which inhibits the activity of adenylate cyclase, reduces the generation of intracellular cyclic adenosine monophosphate (cAMP), prompts the closure of cyclic adenosine-gated ion-controlled channels, and inhibits Na^+^ inflow. G_βγ_ not only inhibits L-type voltage-gated calcium channels and prevents Ca^2+^ inflow and neuronal depolarization but also activates G protein-gated inwardly rectifying potassium channels (GIRK), promotes K^+^ efflux and hyperpolarization, attenuates neuronal excitability, and inhibits neurotransmitter release.^[10,11]^ In addition, opioid receptors can mediate different physiological actions by participating in receptor desensitization, internalization, and recovery through the activation of the β-arrestin pathway parallel to the G protein signaling pathway^.[9]^

## 3. Possible mechanisms underlying OIRD

Studies have shown that the major neural nuclei associated with respiration are located in the brainstem, where they are distributed continuously and interact with each other, extending from the pons anastomosed to the caudal medulla, spinal cord, and cranial motor nerves. The central structures that form rhythmic respiratory movements originate mainly from the pre-Bötzinger complex (PBC) in the ventrolateral medulla.^[12, 13]^ PBC injection with opioid agonists in adult rats inhibits respiratory rate, and naloxone injection in the PBC region partially reverses RD induced by remifentanil in rabbits, suggesting the importance of PBC in opioid-induced respiratory rate inhibition.^[14,15]^ It has been shown that OIRD is caused by the activation of opioid receptors on the surface of brainstem respiratory CNS neurons^[16]^ located in the cortex. Peripheral opioid receptor activation may promote RD by reducing wakefulness and sensory drive,^[17,18]^ and agonism of rostral ventral respiratory group opioid receptors can lead to reduced respiratory rates.^[19,20]^ Topical application of the opioid receptor agonist DAMGO to the caudal nucleus tractus solitarii attenuates hypercapnia and hypoxic ventilatory responses, as well as the broncho-lung C-fiber reflex.^[21]^ Injection of DAMGO into the pontine Kölliker-Fuse (KF) nucleus decreases respiratory rate and tidal volume in rats, a mechanism whereby DAMGO activates GIRK channels, leading to hyperpolarization of specific cell populations in the KF nucleus.^[22,23]^ Knockdown of opioid receptors in KF/parabrachial neurons abrogates the inhibition of respiratory rate by morphine (20 mg/kg).^[24]^ In addition, chemoreceptors located in the cortex, center, and periphery are involved in the regulation of respiratory movements. For example, central chemoreceptors located in the ventral region of the brainstem medulla, including the parietal nucleus, macula, and lone bundle nuclei, regulate respiration by sensing changes in ph. Opioids act on central chemoreceptors to suppress the ventilatory response to hypercapnia, which is antagonized by naloxone.^[25]^ Glomus cell type I, located on the peripheral chemoreceptors of the carotid artery at the common carotid bifurcation, is sensitive to oxygen and carbonic acid and improves respiration by rapidly sensing changes in CO_2_, O_2_, and pH in the blood and cerebrospinal fluid.^[26]^ Studies have shown that enkephalin can inhibit the carotid body, while naloxone can enhance the carotid body and hypoxic ventilatory responses.^[18,27]^

## 4. Clinical symptoms of OIRD

Opioid overdose can trigger RD, beginning with slow, shallow, and irregular respiration, followed by periodic breathing, and ending with rhythmic cessation of respiratory activity manifested by hypercapnia, hypoxemia, and reduced frequency (<8/min) and pauses, leading to cardiopulmonary arrest and even death.^[28]^ In addition, opioids can cause stiffness in respiratory skeletal muscles, including the diaphragm, chest wall, and upper respiratory tract muscles,^[29]^ which may accelerate opioid overdose-related acute death.^[29]^ In rodents, rigidity can lead to the “Straub phenomenon” in animals, which presents a rigid “S” shape — back divergence, vertical tail, and extended paralysis of the hind limbs.^[30]^ Therefore, in patients under anesthesia with high-dose fentanyl injection during cardiac surgery, rigid “wooden chest” and laryngeal spasm may affect mask ventilation. Chest wall or airway obstruction limits the tidal volume, thereby obstructing ventilation and promoting hypoxia. Hypoxia caused by inadequate ventilation is the primary cause of death. Although skeletal muscle rigidity can be attenuated by a combination of the alpha2-adrenergic agonist dexmedetomidine,^[31,32]^ benzodiazepine tranquilizers,^[33]^ and general anesthetics, the combination of opioids with other target sedatives may further aggravate central RD and cause death.^[34,35]^

## 5. Novel opioids

After the opioid receptor is activated, different downstream signaling pathways can activate different cascades, thus eliciting different biological effects. To separate opioid receptor-mediated narcotic analgesia from RD, researchers have focused on novel opioid medications such as mixed opioid receptor agonists, peripherally selective opioid receptor agonists, and opioid receptor splice variant agonists.

### 5.1. Mixed opioid receptor agonists

Studies have shown that mixed opioid agonists can effectively reduce RD and other side effects.^[36,37]^ For example, nalbuphine and dezocine, which are commonly used clinically, are MOR/KOR partial agonists and both cause weaker RD.^[38,39]^ The diarylmethylpiperazine compound DPI-125, a mixed agonist of the opioid receptor, exhibits strong analgesic properties and a high respiratory safety profile in a rat tail-flick pain model.^[40]^ MP1104, a DOR/KOR agonist, attenuated mechanical and cold pain in a paclitaxel-induced mouse model of neuralgia, and unlike morphine, did not produce RD in mice.^[41]^ AT-121 is an nociceptin/orphanin FQ (N/OFQ) peptide receptor/MOR-partial agonist, and both the NOP antagonist J-13397 and the MOR antagonist naltrexone antagonize it, providing 100 times more analgesia than morphine in adult rhesus monkeys, but has no effect on the respiratory rate and ventilatory in rhesus monkeys and does not cause common adverse effects of opioids.^[42,43]^ The naltrexone analog BU10038 is a MOP/NOP partial agonist, and compared with morphine, systemic administration of BU10038 has good analgesic activity but no RD, abusive tendencies, or physical dependence.^[44]^ Cebranopadol was found to have high binding affinity for MOR/KOR/NOP receptors in 2014 and was found to be a full agonist for MOR and NOP receptors in ^[35S]^GTPγS function assay,^[45,46]^ while clinical trial healthy volunteers received a single oral dose of cebranopadol 600 mg with a ventilatory volume greater than 0 L/min, while MOR full agonists such as morphine and fentanyl induced apnea at equivalent doses, suggesting weak respiratory suppression and higher safety.^[46,47]^ Therefore, mixed opioid receptor agonists exhibit good analgesic activity and weak effects on RD.

### 5.2. Peripheral selective opioid receptor agonists

Studies have confirmed that opioid-induced RD is primarily driven by the activation of central opioid receptors and analgesia, primarily by the activation of opioid receptors on peripheral sensory neurons, and selective agonists of peripheral opioid receptors may lead to weaker RD.^[48]^ The fluorinated fentanyl derivative NFEPP has been reported to have a dissociation constant pKa of 6.7, which is close to the pH of inflammatory tissues (5–7) but distant from that of normal tissues (7.4), resulting in more peripheral selectivity.^[49,50]^ In radioligand-binding experiments, NFEPP competed with the radiolabeled endogenous ligand [^3^H]DAMGO at the binding site, suggesting that NFEPP may bind to the MOR. In persistent and acute inflammatory rat pain models, low-dose NFEPP produced dose-dependent analgesia only in inflamed injured paws, whereas fentanyl produced analgesia in both the inflamed and healthy contralateral paws. Fentanyl causes RD; however, NFEPP was not evident.^[51]^ Jimenez-Vargas et al^[50]^ also demonstrated that in colitis mice, NFEPP preferentially activates MOR located in inflamed, acidified tissues, inducing an anti-inflammatory response without RD. Naloxonemethiodide, an MOR antagonist that fails to penetrate the blood-brain barrier but antagonizes the antinociception of NFEPPs, partially reverses the antinociception of fentanyl,^[52,53]^ which further suggests that NFEPPs may act only to produce less RD in the MOR of injured peripheral tissues.

### 5.3. Opioid receptor splicing variants agonists

The MOR gene undergoes extensive selective splicing in vivo, which occurs during the conversion of DNA to messenger RNA by binding to exons that generate multiple receptor subtypes.^[54]^ The 1 transmembrane (1TM) splicing variants contain only exon 1, the 6TM splicing variants contain exon 11, but not exon 1, and the 7TM full-length domain splicing variants containing exons 11 and 1 are classified according to their transmembrane structure. Most receptors present in vivo are 7TM.^[55]^1TM does not bind directly to opiates and indirectly enhances the expression and pharmacological action of 7TM mainly via chaperone proteins.^[56]^ Several opioids targeting 6TM have been characterized as strong analgesics and weak RD.^[57]^ Radiolabeled 3-iodobenzoyl-6beta-naltrexamide (IBNtxA) binding sites were detectable in the meningeal homogenates of wild-type mice and mice lacking 7TM splice variants but not in exon 11 knockout mice, suggesting that IBNtxA may be a novel MOR agonist in 6TM splice variants,^[58]^ further studies confirmed that IBNtxA has high affinity and activity with MOR.^[59]^ Intravenous administration of IBNtxA can achieve analgesic activity similar to that of morphine in hot-plate hyperalgesia experiments in C57 mice.^[60]^ Majumdar et al^[61]^ further demonstrated that subcutaneous IBNtxA (2.5 mg/kg, n = 5) inhibited the respiratory rate in normal mice significantly less than morphine (20 mg/kg, n = 5), and showed no significant difference from saline in the respiratory rate in normal mice (*P *< .001), suggesting that IBNtxA may have the advantages of strong analgesia and weak RD; however, clinical data are lacking for IBNtxA.

### 5.4. Biased opioid receptor agonists

In recent years, researchers have found that different ligands can activate the same receptor but produce different signal transduction cascades that result in different functional outcomes.^[62]^ As far as the opioid system is concerned, opioids may activate G-protein-mediated signaling pathways, a parallel signaling pathway mediated by β-arrestin may also be activated simultaneously. Different ligands may have different levels of activation in these 2 pathways, and may even be biased to activate only one of these pathways, thus exhibiting different biological effects at the overall animal level, such as analgesia, euphoria, and somatic dependence mediated mainly by G-protein signaling pathways and RD mediated by β-arrestin^[63,64]^; Schmid et al^[65]^ showed that ligand bias factors on G-protein signaling were associated with their therapeutic window, suggesting that if ligands were biased to activate the G-protein signaling pathway, RD might be greatly attenuated while producing analgesia. Therefore, over the past decade, one focus of novel opioids has been the design of biased MOR agonists with a G protein bias.^[66]^ Novel opioid receptor agonists such as TRV-130^[67]^ and PZM21,^[68]^ which are thought to have G-protein-biased, low β-arrestin recruitment agonists, may have high analgesic and weak RD potential. TRV130 is a crystal structure screening based on MOR by Trevena Corporation and, compared to morphine, only about 15% of TRV130 is biased toward the β-arrestin pathway by Path Hunter enzyme complementary assay and about 84% of TRV130 is biased toward the G protein signaling pathway by cAMP cumulative assay with G protein bias.^[69]^ In hot-plate experiments in mice and rats, TRV130 demonstrated rapid analgesia without significant RD.^[70]^ Clinical trial results also showed that the analgesic effect of TRV130 was comparable to that of morphine, but had a faster onset, with a lower rate and weaker degree of RD. Recently obtained FDA approval has been shown to have a better safety profile than morphine in humans, TRV130 and morphine were equianalgesic with similar potency values. A 50% reduction of the hypercapnic ventilatory response by morphine occurred at 33.7 ± 4.8 ng/mL, while a 25% reduction by TRV130 occurred at 27.4 ± 3.5 ng/mL (*P* < .01).^[71]^

### 5.5. Allosteric modulator of opioid receptors

In contrast, allosteric ligand-binding sites are different from normal MOR sites, which may have some advantages in spatiotemporal control of receptor activation and behavior. Allosteric modulators, particularly positive allosteric modulators (PAM), can enhance the affinity, potency, and efficacy of exogenous drugs or endogenous opioid peptides, while negative allosteric modulators can inhibit the binding of normal ligand agonists and/or decrease the functional activity of ligands^[72,73]^ Silent allosteric modulators do not directly affect the binding or activity of orthotopic agonists but block the activity of negative or orthostatic modulators.^[16]^ Based on this theory, PAM can be combined with classical orthostatic sites with opioid analgesics, such as morphine, fentanyl, or oxycodone, to reduce the dose of opioid analgesics, thereby reducing RD and making it safer.^[74]^ Furthermore, the PAM of the MOR promotes the activity of endogenous opioid peptides released during pain,^[72,75]^ replacing traditional opioids by enhancing the natural response of the human body to pain and inducing a temporary and local analgesic response at the site of the onset of pain in the body, thereby providing clinical analgesia. BMS-986122 was recently identified as a PAM in MOR that enhances endorphin signaling via a Na^+^ regulatory site that binds to MOR.^[76]^ BMS986122 is an analgesic in the absence of exogenous opioid administration and has no side effects, such as constipation, RD, or reward.^[77]^ It has been suggested that PAM of the MOR, as a potential new mechanism for the treatment of interventional pain, may contribute to the reduction of side effects such as RD.^[77]^

### 5.6. Others

Different subtypes of opioid receptors can form dimers or oligomers coupled with other G protein receptors, which exhibit pharmacological properties that are different from those of a single receptor when activated. MOR/DOR heterodimers have been confirmed to be co-expressed in cell cultures in vitro.^[78]^ Some studies have confirmed that 6’-guanidinonaltrindole can activate DOR/KOR heterodimers, exhibit different signaling and functional regulatory effects, and exert analgesic effects without adverse effects such as RD.^[79,80]^ In addition, it has been demonstrated that different subtypes of opioid receptors mediate different degrees of RD and analgesia after activation, such as when given the selective MOR agonist DAMGO, which can produce significant RD, whereas when given the selective DOR agonist U-50488H, RD is weak.^[81]^ Studies have also confirmed that endogenous endorphins are highly selective for MOR, and compared with conventional exogenous opioids, endorphin analogs are effective in reducing the incidence of receptor downregulation, tolerance, and desensitization and have weaker RD.^[82]^ Therefore, the development of endorphin analogs or enkephalin inhibitors is promising, such as in a hot-plate hyperalgesia mouse model, where the analgesic effect of the endorphin-1 analog ZH853 is comparable to that of morphine. However, ZH853 showed a significantly lower RD than morphine in SD rats.^[83]^ Considering the complexity of compound synthesis and the lack of safety and efficacy data, no relevant clinical trial studies have been reported.

## 6. Non-opioid to improve OIRD

Reversal of RD through pathways other than opioid receptors and prevention of RD without affecting the efficacy of opioids themselves may be a feasible strategy that could have a broad spectrum of respiratory stimulants, with more non-opioid research currently focusing on the following targets:

### 6.1. AMPA receptor modulator

The AMPA receptor is an ionotropic transmembrane receptor activated by glutamate which mediates most of the rapid synaptic excitatory neurotransmission distributed throughout the central nervous system. When activated, it promotes glutamate influx, leading to respiratory excitation, and is important for regulating the respiratory rhythm.^[84,85]^ RespireRx has developed a series of benzamide AMPA receptor-positive allosteric modulators, including CX546 and CX717, which have entered clinical phase II for the treatment of neurological disorders such as Alzheimer’s disease, attention deficit hyperactivity disorder, and cognitive dysfunction.^[86]^ Preclinical and clinical studies have demonstrated good anti-RD activities and enhanced analgesic effects. In rats, fentanyl-induced RD was reversed by CX546 and CX717, which reversed or prevented fentanyl-induced deep RD and apnea.^[87]^ In healthy men, CX717 in combination with alfentanil significantly increased blood oxygenation levels and produced a greater ventilatory response to hypercapnia without interfering with alfentanil-induced analgesia.^[88]^ CX546 in combination with morphine produced additional analgesia in postoperative naïve and adult rats.^[89]^ Tianeptine, an antidepressant and cognitive-enhancing agent, phosphorylates AMPA receptors, prevents morphine-induced RD in pretreated rats, and increases respiratory rate, tidal volume, and minute ventilation without affecting analgesia.^[90]^ A recently reported novel AMPA modulator, LCX001, which increases respiratory rate and minute ventilation in rats, prevents RD induced by fentanyl and dose-dependently increases analgesia in rats.^[91]^ As AMPA modulators may also affect the nervous system while regulating respiration, the regulation of the dual system may reduce its clinical selectivity and safety.

### 6.2. The 5-hydroxytryptamine (5-HT) receptor agonist

5HT receptors, including receptor subtypes 5-HT1A, 5-HT2A, 5-HT2B, 5-HT2C, 5-HT4, and 5-HT7, are widely expressed in the brain stem and are involved in regulating a variety of physiological responses, including breathing after activation.^[85,92]^ 5-HT1A, 5-HT7, and 5-HT4 receptors are potential targets of ORID. Studies have shown that the selective 5-HT1a receptor agonist 8-OH-DPAT and 5-HT4 receptor agonist/5-HT3 antagonist zacopride (Zacopride) reversed etorphine-induced goat RD, and the 5-HT1a receptor-partial agonist buspirone and the selective 5-HT4a receptor agonist BIMU8 reversed fentanyl-induced RD and apnea in rats with no effect on analgesia.^[93]^ Repinotan, a selective 5-HT1a agonist, averted OIRD while prolonging analgesia in rats, The ventilatory depression by remifentanil induced (2.5 µg/kg; A 65% reduction; *P* = .031, n = 5) was attenuated by repinotan (20 µg/kg; A 24% reduction; *P* = .313, compared with the pretreatment level).^[94,95]^ Manzke et al^[96]^ confirmed that 5-HT1a agonists can inhibit inhibitory neuronal glycine, leading to an increase in glycine-mediated neural excitability, which reverses opioid-induced RD. However, the commonly used antidepressant drug buspirone^[97]^ and the 5-HT4a receptor agonist motility-stimulating drug mosapride^[98]^ do not improve opioid-induced human RD, which may be due to poor pharmacokinetics, inadequate brain penetration, and low drug potency. Recent studies have also found that activation of 5-HT1a receptors located in the PBC and ventral respiratory columns has little effect on respiratory stimulation.^[99]^ Therefore, the regulatory role of 5-HT receptors in the respiratory function remains an important research topic.

### 6.3. Nicotinic acetylcholine receptor agonist

The medulla, including the PBC respiratory neurons, expresses the nicotinic acetylcholine receptor, which is symmetrically arranged around one central foramen by 5 subunits (α4, α7, and β2) located in the medullary respiratory network, and activated receptors can stimulate respiration. Ren et al^[100]^ found that the α4β2 nicotinic acetylcholine receptor agonist A85380 can quickly reversed RD and apnea induced by opioids in rats and synergistically increased the analgesic effect. Administration of A85380 (0.03 mg/kg), provides a rapid reversal of fentanyl induced decrease in respiratory rate (93.4 ± 33.7% of control after A85380 vs 31 ± 20.5% of control after vehicle, n = 8, *P* < .001) in adult rats, without obvious side effects. The water solubility of A85380 was similar to that of naloxone, with a half-life of about 7 hours, whereas the naloxone half-life was 30 minutes; further studies found that the α4β2 nicotinic acetylcholine receptor partial agonists, Varenicline and ABT594 improved the effects of fentanyl on moderate to severe RD in rats without affecting analgesia, whereas ABT594 also synergistically promoted fentanyl analgesia against lethal doses of fentanyl or deaths caused by fentanyl-diazepam co-administration.^[101]^ To date, there have been no corresponding clinical data studies.

### 6.4. Phosphodiesterase-4 (PDE-4) inhibitors

It has been shown that RD is caused by opioids reducing cAMP content in respiratory-related neurons, and the pharmacological effects of methylxanthines, such as caffeine and aminophylline, may stem mainly from the blockade of the brainstem respiratory center PDE-4 receptor, activating intracellular cAMP – Protein-Kinase-A signals, elevating cAMP content in the central nervous system,^[102]^ stimulating respiratory rhythm formation, and exciting breathing. For example, the selective PDE-4 inhibitor rolipram can restore fentanyl-induced RD in rats without affecting analgesia^[103]^; however, no corresponding clinical report has been reported. Caffeine is a nonselective PDE-4 inhibitor with adenosine antagonistic activity that is used clinically for the treatment of apnea and chronic obstructive pulmonary disease in preterm infants. A 65-year-old man was confirmed to have induced apnea after receiving remifentanil, and subsequently recovered breathing after 5 mg caffeine.^[104]^ However, methylxanthines may have off-target effects, causing changes in coronary flow and heart rate, increasing seizures, and, at high doses, affecting brainstem development and decreasing survival from hypoxia in premature infants. Therefore the safety requires further investigation.

### 6.5. K^+^ channel blocker

The respiratory center of the brainstem is regulated at several sites, including the oxygen-sensitive K^+^ channel of the peripheral chemoreceptor type I glomus cells of the carotid artery located at the common carotid bifurcation. It has been shown that direct activation of neuronal K^+^ channels located in the PBC and sublingual nerve nuclei in mice stimulates respiration,^[105]^ but its clinical application is limited to a greater extent owing to the presence of side effects such as headache, nausea, and anxiety. GAL-021 can stimulate respiration by blocking the large conductive calcium-activated K^+^ channel in the carotid body, which is significantly reduced by carotid sinus nerve resection in rats,^[106]^ and by a dose-dependent increase in minute ventilation, which improves RD in rats and humans with morphine and fentanyl, without affecting analgesia.^[107]^ However, the short duration of action of GAL-021, approximately 5 to 10 minutes, and the upper limit effect of the improvement in breathing did not effectively improve deep RD and apnea induced by opioids, and it may be difficult to overcome severe RD originating in the center by stimulating peripheral chemoreceptors.^[108]^

### 6.6. Others

Studies have shown that non-opioid drugs, such as the dopamine D1 receptor agonist dihydrexidine,^[109]^ thyrotropin-releasing hormone and its analog taltirelin,^[110]^ N-methyl-D-aspartate receptor antagonist ketamine,^[111]^ glial cell inhibitor minocycline,^[112]^ cholinesterase inhibitor toluene, donepezil,^[113]^ glycine-glutamine,^[114]^ and orexin^[115]^ can also improve breathing without affecting or promoting opioid analgesia. However, owing to the large number of side effects or insufficient clinical data, the safety and efficacy of ketamine can cause tachycardia, nausea, hallucinations, vomiting, and anxiety, which limits its further use.

## 7. Conclusion

Activation of opioid receptors located at sites such as the PBC, nucleus tractus solitarius, and KF nucleus is an important cause of RD produced by opioids, probably through mechanisms such as inhibition of adenylate cyclase, GIRK channels, and voltage-gated calcium channels that regulate respiration. However, the regulation of respiratory movement is complex; opioid receptors are widely distributed, and the effects of opioids acting at different neural sites on respiration have not been fully elucidated. Considering that OIRD is a serious complication of opioids, timely detection and correction of OIRD can still be achieved with surveillance when high-dose opioids are used in clinical anesthesia. However, not every case of RD is timely detectable in the management of acute and chronic pain and must be given sufficient attention. In recent decades, many researchers have attempted to develop novel drugs that can replace opioids; however, most patients with chronic pain cannot achieve complete pain relief with first-line treatments, such as calcium channel inhibitors (e.g., gabapentin and pregabalin), antidepressants, and/or anticonvulsants, and patients tend to be unable to tolerate effective doses owing to serious adverse effects.^[16]^ Therefore, improving opioids, developing novel opioids for weak RD, and improving RD by using non-opioid drugs are current research hotspots. We must fully explore each approach and expand our basic scientific knowledge on all levels of pain, analgesia, and breathing. Novel opioid and non-opioid drugs can improve and relieve OIRD from different perspectives, mechanisms, and routes; effectively improve the safety of clinical opioid applications; and open up a larger field of vision and updated ideas and understanding for clinical treatment. Therefore, both these concepts are scientifically and clinically significant. The long-term safety and efficacy of the novel candidate drugs mentioned in this review require further evaluation. It is believed that with in-depth research on opioid receptor structures, signaling pathways, and respiratory targets, novel opioids with strong analgesic effects, small side effects of RD, or non-opioid drugs that do not affect analgesic activity and even have synergistic analgesic activity will be expected in the future, which will reduce the incidence of RD during surgery and improve the safety of clinical medications.

## Author contributions

**Conceptualization:** Yong-Zheng Fan, Yun-Li Duan.

**Formal analysis:** Chuan-Tao Chen, Yu Wang.

**Project administration:** An-Ping Zhu.

**Writing – original draft:** Yong-Zheng Fan, Yun-Li Duan, Chuan-Tao Chen.

**Writing – review & editing:** Yu Wang, An-Ping Zhu.
